# Broadband Optical Activity Spectroscopy with Interferometric Fourier-Transform Balanced Detection

**DOI:** 10.1021/acsphotonics.0c01866

**Published:** 2021-07-13

**Authors:** Soumen Ghosh, Georg Herink, Antonio Perri, Fabrizio Preda, Cristian Manzoni, Dario Polli, Giulio Cerullo

**Affiliations:** † Dipartimento di Fisica, Politecnico di Milano, Piazza Leonardo da Vinci 32, I-20133 Milano, Italy; ‡ Experimental Physics VIII, University of Bayreuth, D-95447 Bayreuth, Germany; § NIREOS S.R.L., Via G. Durando 39, 20158 Milano, Italy; ∥ Istituto di Fotonica e Nanotecnologie (IFN)−CNR, Piazza Leonardo da Vinci 32, I-20133 Milano, Italy

**Keywords:** chirality, optical activity, circular dichroism, heterodyne spectroscopy, balanced detection, time domain interferometry

## Abstract

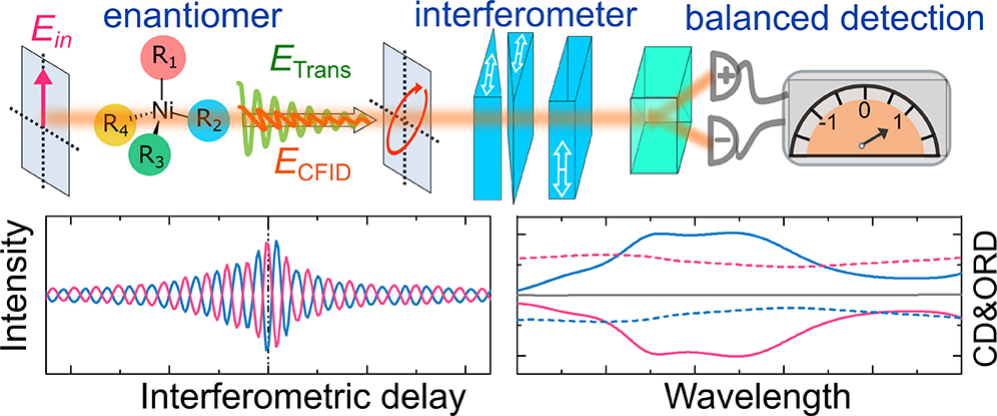

Spectrally resolved measurements of optical activity, such as circular dichroism (CD) and optical rotatory dispersion (ORD), are powerful tools to study chiroptical properties of (bio)molecular and nanoplasmonic systems. The wider utilization of these techniques, however, has been impeded by the bulky and slow design of conventional spectropolarimeters, which have been limited to a narrowband scanning approach for more than 50 years. In this work, we demonstrate broadband measurements of optical activity by combining a balanced detection scheme with interferometric Fourier-transform spectroscopy. The setup utilizes a linearly polarized light field that creates an orthogonally polarized weak chiral free-induction-decay field, along with a phase-locked achiral transmitted signal, which serves as the local oscillator for heterodyne amplification. By scanning the delay between the two fields with a birefringent common-path interferometer and recording their interferogram with a balanced detector that measures polarization rotation, broadband CD and ORD spectra are retrieved simultaneously with a Fourier transform. Using an incoherent thermal light source, we achieve state-of-the-art sensitivity for CD and ORD across a broad wavelength range in a remarkably simple setup. We further demonstrate the potential of our technique for highly sensitive measurements of glucose concentration and the real-time monitoring of ground-state chemical reactions. The setup also accepts broadband pulses and will be suitable for broadband transient optical activity spectroscopy and broadband optical activity imaging.

The field of chiroptical spectroscopy is undergoing a renaissance due to the growing need for table-top optical spectroscopies with exquisite structural sensitivity in the condensed^1,2^ and gas phases^3,4^ and to the emerging field of plasmonic chirality.^5,6^ Optical activity (OA) describes the interaction of chiral enantiomers with polarized light and manifests in circular dichroism (CD) and optical rotatory dispersion (ORD), which measure respectively the different absorption and refraction of left (LCP) and right (RCP) circularly polarized light. CD spectroscopy is performed in resonance with electronic/vibrational transitions and is routinely employed to determine the handedness and structural organization of chemical, biological, and material systems.^7,8^ ORD, on the other hand, has the advantage that it is nonzero outside the absorption band and can distinguish chiral molecules even in nonresonant conditions. Both CD and ORD spectra are enantio-differentiating in their sign and can be correlated to the absolute molecular configurations in condensed phases through ab initio quantum-chemical calculations.^9,10^

CD and ORD represent the imaginary and real parts of a complex chiroptical susceptibility, just as ordinary absorption and dispersion correspond to the imaginary and real parts of the complex refractive index. As such, they are related by a Kramers–Kronig transform; yet their measurement typically requires two separate optical setups. For over 50 years, CD spectrometers and ORD polarimeters have relied on fast polarization switching and synchronous lock-in detection. A CD spectrometer typically uses a photoelastic modulator (PEM), working at a tens of kHz repetition rate, to switch a linearly polarized monochromatic light between RCP and LCP, and synchronously detects the small absorption difference by a lock-in amplifier. Polarimeters use a similar setup to measure ORD, but the PEM is configured as a half-waveplate to switch between orthogonal linearly polarized light components, with an analyzer placed between the sample and the photodetector. The optical rotation at a specific wavelength is inferred from the change in the light intensity transmitted by the analyzer at twice the PEM modulation frequency. Wavelength scanning of a broadband source with a monochromator allows then the reconstruction of CD/ORD spectra. Although standard spectropolarimeters offer high sensitivity, their scan rate is quite low due to the narrowband serial approach, which requires long acquisition times.

Measurements of broadband CD and ORD spectra with high sensitivity have been challenging, as chiral signals are very weak in comparison to achiral ones. CD, for instance, is 3–5 orders of magnitude smaller than absorbance and requires a detection sensitivity on the order of millidegrees in units of ellipticity, where 1 mdeg ≈ 3 × 10^–5^ optical density. Such high sensitivity is difficult to achieve in standard setups using a differential measurement and a multichannel detector, in which the read-out rate is limited to the kHz regime. Furthermore, the wavelength-dependent polarization behavior of optical components can introduce significant artifacts in the measurement, thus distorting broadband CD/ORD spectra. Only a few experimental configurations for broadband electronic OA spectroscopy have been demonstrated thus far, mostly using coherent ultrafast laser sources. One such approach employs kHz repetition-rate ultrafast lasers with a broadband polarization modulation scheme to switch between RCP and LCP pulses and shot-to-shot intensity measurement on a CCD/CMOS detector.^11−13^

Cho and co-workers have introduced an electric-field-based approach for the interferometric characterization of the chiral light field.^14^ In this method, a linearly or elliptically polarized laser beam interacts with a chiral sample to generate an orthogonally polarized chiral signal field, which is then separated in a cross-polarized detection geometry and characterized by heterodyned spectral interferometry. The heterodyne detection strongly amplifies the weak chiral signal by a local oscillator (LO) and fully resolves the chiral signal in amplitude and phase, thus enabling simultaneous measurement of broadband CD and ORD spectra. High phase stability of the interferometer is critical for its successful implementation and requires the use of spectral referencing detection^15,16^ and common-path interferometric configuration.^17^ Recently, we have demonstrated a time-domain interferometric scheme for heterodyne characterization of the chiral signal using a Fourier-transform (FT) approach, which is compatible with coherent as well as incoherent illumination.^18^ Compared to spatially coherent light sources, however, the use of incoherent light requires dedicated optical schemes to preserve the interferometric contrast; as a result, high sensitivity measurement of broadband CD and ORD spectra using incoherent light sources is challenging and has thus far remained elusive.

In this work we introduce an approach to the sensitive measurement of broadband optical activity, which combines a noise-canceling balanced detection scheme with interferometric FT spectroscopy. In our method a broadband light field with a highly pure linear polarization illuminates the chiral sample; optical activity generates a weak chiral field with linear polarization perpendicular to that of the driving field, which causes a slight rotation of the polarization of the transmitted achiral field. We measure such rotation by a balanced photodetector, which ensures high sensitivity through common-mode noise cancellation. Finally, by changing the relative time delay between the chiral and achiral components with interferometric precision and recording the delay-dependent polarization rotation, we retrieve after FT broadband CD and ORD spectra. We demonstrate the high sensitivity and speed of our setup, its broad spectral coverage, and its capability for real-time ground-state reaction monitoring.

## Results

**Figure 1:**
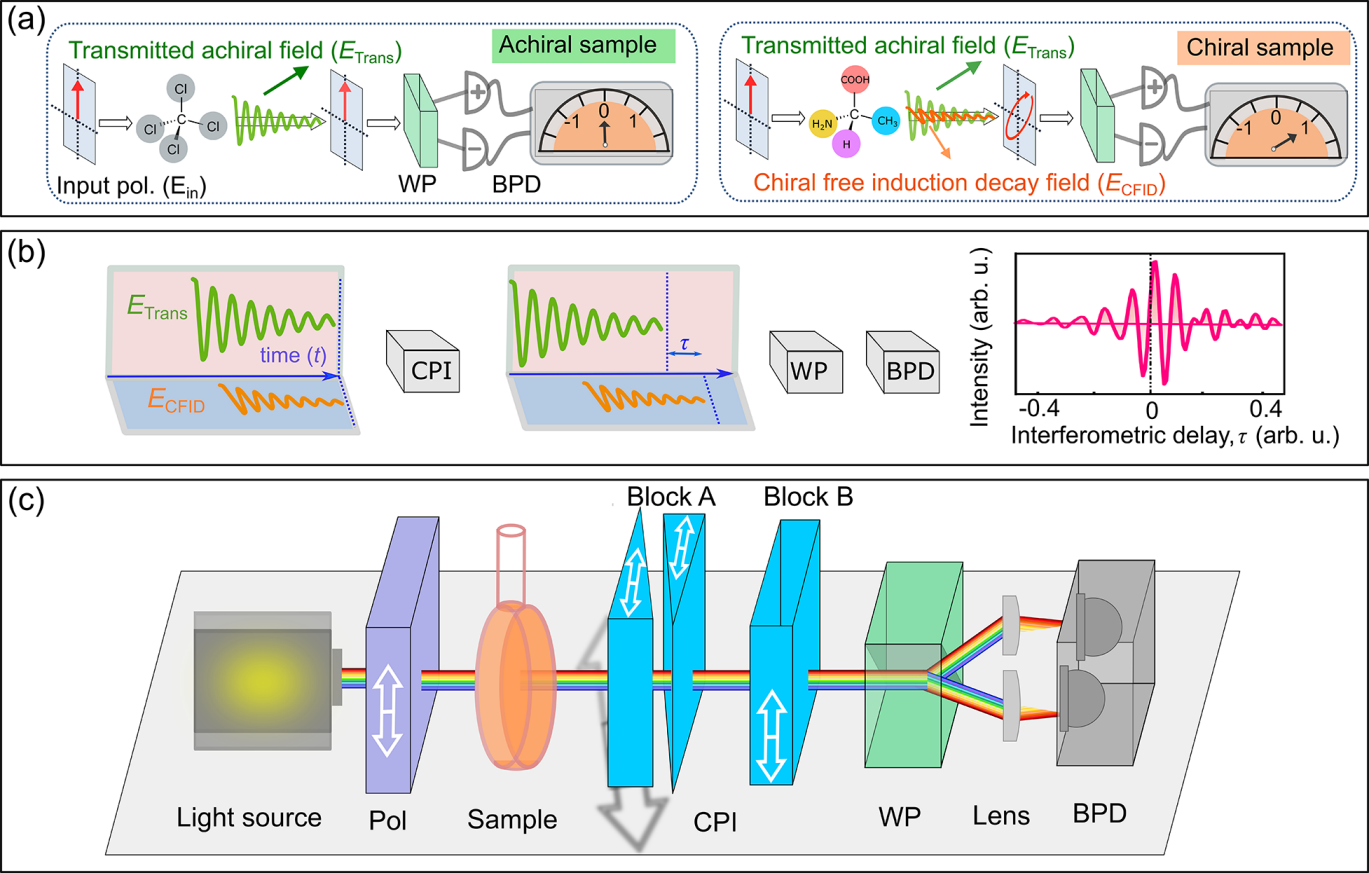
Principle and experimental setup for broadband measurement of optical activity with balanced detection. (a) Balanced detection scheme for the sensitive measurement of polarization rotation. (b) Time-domain interferometry for the broadband measurement of optical activity. Interference of the chiral free induction decay field (*E*_CFID_) and achiral transmitted light (*E*_Trans_) is recorded by a balanced photodetector (BPD) with a time-integrated detection as a function of the delay introduced by a common-path interferometer (CPI). (c) Experimental setup for the balanced detection broadband optical activity measurement. Pol: polarizer; CPI: common-path interferometer; WP: Wollaston prism; BPD: balanced photodetector. The white double arrows indicate the optical axes of the polarization optics. The gray double arrow indicates the direction of the wedge translation.

Figure 1 shows the conceptual scheme and the experimental setup of our interferometric broadband optical activity spectrometer with balanced FT detection. The essence of our approach is a highly sensitive measurement of polarization rotation thanks to the combination of (i) balanced detection; (ii) broad spectral coverage afforded by the FT time-domain interferometry; and (iii) intrinsic interferometric stability of the common-path heterodyne detection scheme. When a linearly polarized light (*E*_in_) interacts with an achiral sample, it creates a time-varying polarization within the sample, which emits an achiral free induction decay (AFID) field, *E*_AFID_, with the same polarization as the incident light (Figure 1a). The transmitted achiral field, which is the superposition of the input and AFID fields (*E*_Trans_ = *E*_in_ + *E*_AFID_), is responsible for linear absorption/refraction. In the presence of a chiral sample, conversely, the linearly polarized input field *E*_in_ creates an additional chiral free induction decay (CFID) field, *E*_CFID_, with perpendicular polarization (horizontal, Figure 1a). The presence of the new CFID with perpendicular polarization results in an output field with elliptical polarization. To detect the change of the polarization state of the output field, we employ a balanced detection configuration, also known as the “optical bridge”.

Our balanced detection scheme,^19^ inspired by the electro-optic sampling approach traditionally used with THz^20,21^ and mid-IR fields,^22,23^ consists of a Wollaston prism (WP) and a balanced photodetector (BPD) comprising two photodiode channels followed by a differential amplifier. The WP separates the light into two beams with orthogonal polarizations, individually measured by the channels of the BPD. The WP is oriented at ±45° with respect to *E*_Trans_ so that, with an achiral sample, *E*_Trans_ (vertical in Figure 1a) is projected into two equal orthogonal components and the BPD registers zero differential signal. Only in the presence of a chiral sample does the resulting *E*_CFID_ unbalance the two projected components, and this results in a finite differential signal from the BPD. Note that the sign of the output signal depends on the direction of rotation of the polarization ellipse, thus providing the enantio-differentiating capability to our setup.

Broadband and phase-sensitive measurement of the CFID is performed with FT time-domain spectroscopy, the principle of which is shown in Figure 1b. A polarization division common-path interferometer^24^ (CPI, see Figure 1c), inserted after the sample, varies the relative delay between the orthogonally polarized achiral and chiral fields. After the CPI, the WP projects the two components into common polarization planes at ±45° and sends them to the BPD. For each delay τ introduced by the CPI, the time-integrated energy measured at each of the channels of the BPD can be written, in the hypothesis in which *E*_CFID_ ≪ *E*_Trans_, as
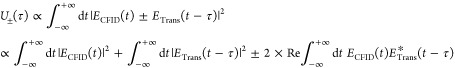
1

The differential output of the BPD, which we call a chiral interferogram, is thus
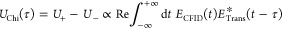
2which is the background-free temporal cross-correlation between the chiral and achiral transmitted fields. Note that the achiral transmitted field, *E*_Trans_(*t*), is much stronger than the weak CFID, *E*_CFID_(*t*). Thanks to the common optical path, it acts as a phase-locked LO. This leads to heterodyne amplification of the chiral signal field, thus enabling its sensitive measurement. The recorded interferogram can be cast into the frequency domain by FT:
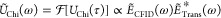
3

An autocorrelation measurement of the transmitted achiral light provides
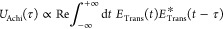
4which, after FT, becomes
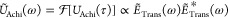
5

The complex chiroptical susceptibility of the medium χ_Chi_(ω), expressed as,^25^
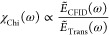
6can be retrieved from the measurements as
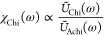
7where the imaginary and real parts of χ_Chi_(ω) correspond to the CD and ORD signals.

Our approach based on the combination of balanced detection with heterodyne time-domain spectroscopy offers several important advantages. As evidenced in eq 1, the interferograms *U*_+_ and *U*_–_ have the same offset and opposite time-correlation functions, due to energy conservation. Thus, the balanced detection setup cancels out the intensity fluctuations of the light source and allows us to measure polarization rotation with very high sensitivity. This aspect, combined with the heterodyne amplification of the weak chiral field by the stronger achiral transmitted field, enables simultaneous measurements of broadband CD and ORD spectra with high sensitivity.

Figure 1c shows the setup of our balanced detection time-domain spectropolarimeter, which works with both temporally coherent and incoherent broadband light sources. We employ a stabilized broadband halogen light source (Thorlabs, SLS201L); after collimation and reduction of the field aperture with an iris, the light is polarized by a high-quality Glan Taylor polarizer (extinction ratio >10^5^), mounted on a high-precision stepper-motor-driven rotation stage. The linearly polarized light travels through the chiral sample, contained in a strain-free cuvette with a 10 mm optical path length (Hellma Analytics, model 120-10-40), and through the CPI, which consists of two birefringent blocks made of α-barium borate (α-BBO) with optical axes orthogonal to each other and to the propagation direction of the beam (see the Methods section for details). By varying the insertion of one of the wedges in the beam path with a micrometer-precision translation stage, one can control the delay between the chiral and achiral fields with very high accuracy (down to ∼1 attosecond), long-term interferometric stability, and reproducibility. After the CPI, the beam passes through the WP (Thorlabs Inc., WP10), with the optical axis oriented at 45°, the two separated orthogonally polarized beams are detected by the BPD (Thorlabs Inc., UV–visible PDB 210A) connected to a data acquisition board. The chiral interferogram *U*_Chi_(τ) is measured by recording the differential signal from the BPD as a function of the insertion of the moving wedge. The transmission axis of the Glan Taylor polarizer with respect to the CPI main axis is finely adjusted in order to cancel any spurious time-domain chiral interferogram in the absence of a chiral sample. Upon rotating the axis of the polarizer from 0° to 45°, we record the achiral interferogram *U*_Achi_(τ), which serves as a calibration to retrieve the broadband CD and ORD spectra according to eq 7. A quantitative calculation of the CD and ORD signals using the Jones matrix formalism is provided in the Supporting Information.

**Figure 2:**
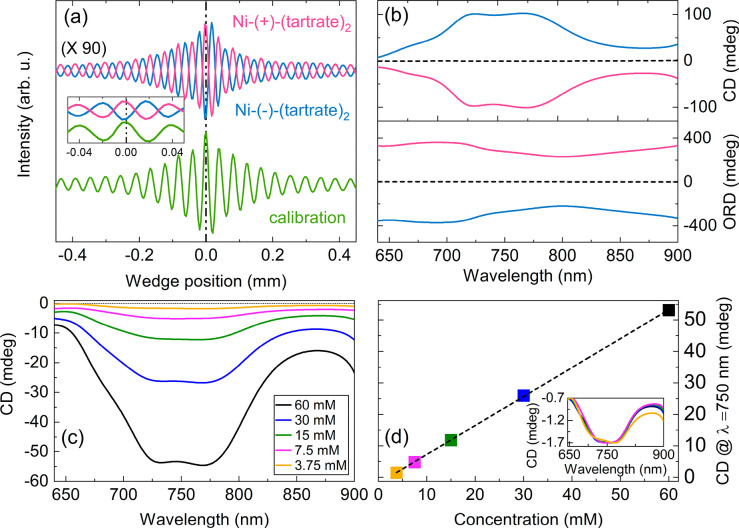
Broadband measurement of optical activity. (a) Chiral (blue and red lines) and calibration (green line) interferograms of a 120 mM aqueous solution of nickel tartrate enantiomers, measured with a halogen lamp and a UV–visible BPD. Inset: zoom-in of the interferograms, (b) Electronic CD (top panel) and ORD spectra (bottom panel) of Ni-(±)-(tartrate)_2_ enantiomers in the visible–near IR region. (c) CD spectra of Ni-(+)-(tartrate)_2_ at different concentrations. (d) CD signal of Ni-(+)-(tartrate)_2_ at 750 nm as a function of concentration. The inset shows the CD spectra of different concentrations normalized by the spectrum of the lowest concentration.

We first validate our approach by measuring the electronic optical activity of the enantiomers of Ni-(±)-(tartrate)_2_ in distilled water. A short-pass filter (FESH0950, Thorlabs) with a cutoff at 950 nm was inserted into the excitation beam path. Figure 2 shows the experimental results obtained with a 120 mM aqueous Ni-(±)-(tartrate)_2_ solution. The enantiomer-dependent phase of the two chiral interferograms is evident in Figure 2a and demonstrates the enantio-differentiating capability of our time-domain approach. The scan of each interferogram takes about 5 s. A small phase shift is also noticeable between the chiral interferograms and the calibration interferogram and is utilized to retrieve CD and ORD from the complex signals obtained after FT of the interferograms. The magnitude of the shift depends on the strength of the CD signals.

Figure 2b shows the broadband CD (in units of ellipticity) and ORD spectra obtained after baseline subtraction with a solvent-filled cuvette. Both spectra have opposite signs for the enantiomers; in addition, CD spectra are consistent with those measured using a commercial CD spectrometer. To test the sensitivity of our setup, we have recorded CD spectra of aqueous Ni-(+)-(tartrate)_2_ at different concentrations. Figure 2c and d show the CD spectra measured at different dilutions and their concentration dependence, respectively. We observe that, as the concentrations are reduced, the CD amplitude scales linearly and spectral line shapes are preserved, as shown in the inset of Figure 2d. These results demonstrate the quantitative capability of our setup for broadband electronic OA measurement using an incoherent light source. The CD detection sensitivity of the setup is better than 3.75 mM, corresponding to an absorptivity smaller than 6 × 10^–5^ (2 millidegrees), sufficient for most applications requiring electronic CD measurements. For very small CD signals (<1 mdeg), however, we observe distortions in the measured spectra due to contributions from polarization artifacts of the optical system. Our current results represent an almost 10-fold improvement in the CD sensitivity compared to our previous approach employing a nearly crossed polarizer geometry.^18^ The noise-canceling ability afforded by the balanced configuration thus enables sensitive measurement of broadband electronic CD spectra using incoherent light sources.

It is worth pointing out that, although in principle one can calculate an ORD spectrum from the Kramers–Kronig transform of a CD spectrum, this is not practically feasible since it requires the acquisition of the full CD spectrum over a broad wavelength range. For this reason, CD and ORD are typically measured independently by different experimental techniques. Our setup offers the unique advantage of performing simultaneous measurement of broadband electronic CD and ORD spectra, in a highly simplified setup without PEMs or lock-in amplifiers and with fast (a few seconds) acquisition times. We are only limited by the dynamic range of the detector (since the detectors also collect the strong achiral signal serving as LO). We expect further improvements in sensitivity by combining balanced detection with near cross-polarizer heterodyne amplification.

**Figure 3:**
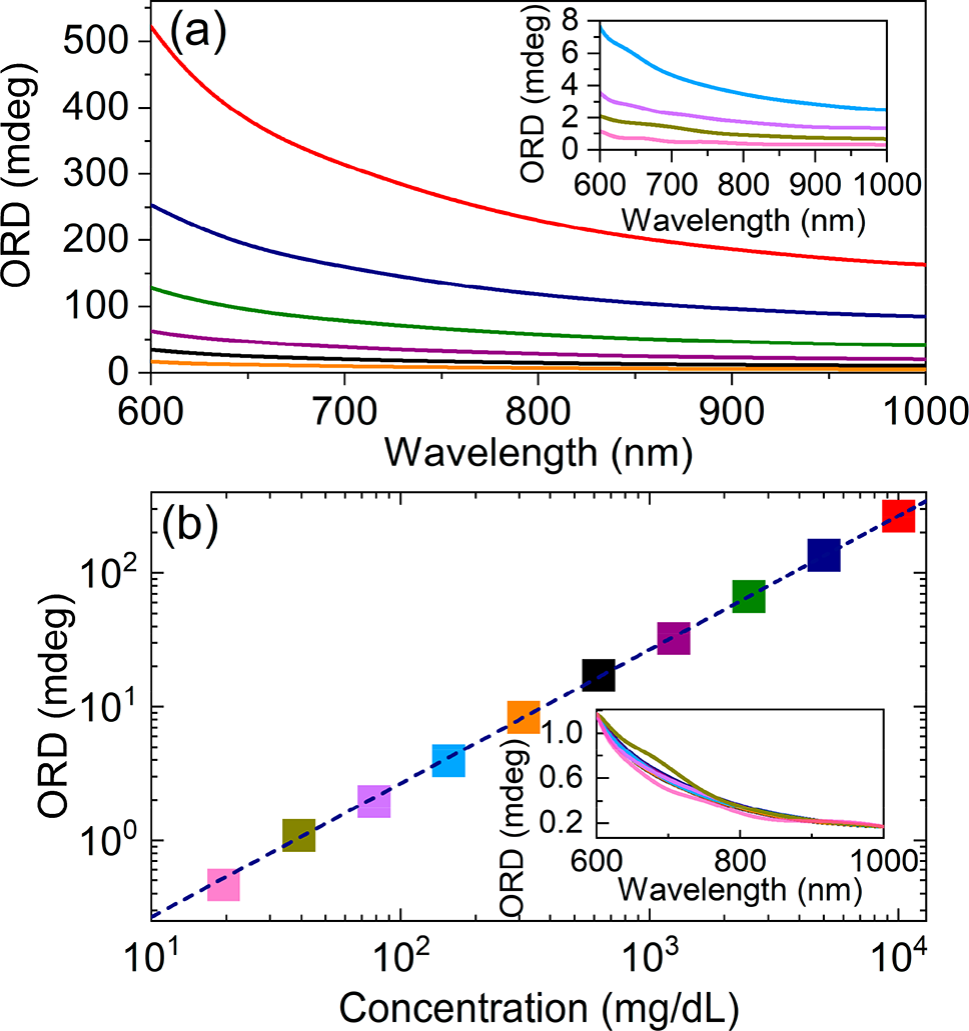
High-sensitivity detection of glucose concentration. (a) ORD spectra of a glucose solution in a 10 mm path-length cuvette measured with a halogen lamp. The inset shows the low concentration spectra with rescaled y-axis (b) ORD signal at 750 nm as a function of glucose concentration. The inset shows the normalized ORD spectra of different concentrations overlaid with the spectrum of the lowest glucose concentration. The same color code has been used to correlate the ORD spectrum with the glucose concentration.

We test the sensitivity of the setup for broadband ORD measurements using an aqueous solution of glucose. Figure 3a and b display the ORD spectra of glucose at different dilutions and the concentration dependence of glucose ORD at 750 nm, respectively. The setup is capable of quantitatively measuring optical rotation down to <0.2 millidegree, which translates into sensitivity better than 3.5 × 10^–6^ radians, comparable to that obtained by commercial narrowband polarimeters. With such sensitivity, we can detect glucose concentrations as low as 20 mg/dL.

**Figure 4:**
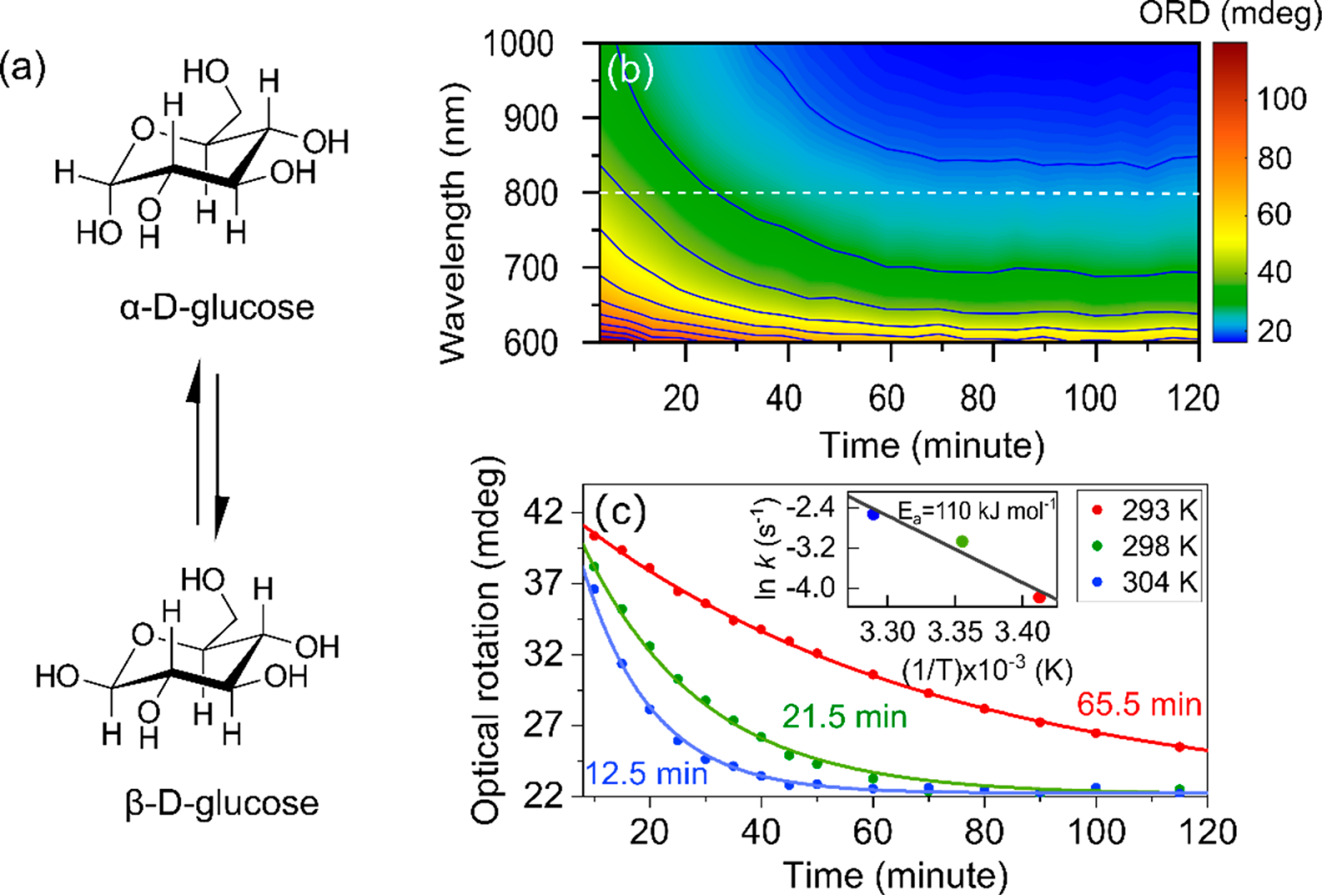
Real-time monitoring of a ground-state chemical reaction. Mutarotation of α-d-glucose measured in a cuvette with a 1 mm path length. (a) Conversion of α-anomer to β-anomer and vice versa. (b) Contour plot of the evolution of ORD spectra as a function of time at 298 K. Each spectrum was acquired with 5 scans of the interferometer, resulting in an acquisition time of less than half a minute. (c) Dynamics of ORD values at 800 nm during mutarotation, at different temperatures. The dots are experimental data, and the solid lines are exponential fits with the given time constants. The inset shows the fitting of the rate constant using the Arrhenius equation.

Thanks to the short acquisition times, our setup can perform in situ monitoring of chemical reactions in real time. An example of a ground-state chemical reaction with a time-dependent change in optical activity is the mutarotation of glucose. In water, α-d-glucose is converted to its anomeric form β-d-glucose, resulting, at equilibrium, in a mixture of about 32% α-anomer and 68% β-anomer (see Figure 4a). Since the β-anomer has smaller optical rotation values than the α-anomer, the progress of mutarotation can be monitored by measuring the ORD spectrum of the mixture at different times. Figure 4b shows the ORD contour plot measured at 298 K after dissolving 100 mg/mL α-d-glucose in distilled water. We started measuring ORD spectra 5 min after adding α-d-glucose, which is sufficient for complete dissolution and for obtaining a thermally equilibrated mixture. As expected, the formation of the β-anomer is accompanied by a gradual decrease in the intensity of the measured ORD spectra, until an equilibrium is reached between the two anomers. Since the optical rotation is directly related to the concentration of α-d-glucose, the progress of the reaction at a particular wavelength can be expressed as
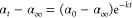
8where *α*_*t*_ and α_0_ are the observed rotations at time *t* and *t* = 0, respectively, and α_∞_ denotes optical rotation after reaching equilibrium. By fitting our data with eq 8, we obtained that the reaction rate is *k* = 7.75 × 10^–4^ s^–1^ at 298 K. We also changed the temperature of the mixture to vary the rate of mutarotation; this is shown in Figure 4c, which clearly displays a faster decay of ORD values at higher temperatures. The dependence of the rate constants on temperature is fitted with the Arrhenius equation
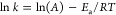
where *A* is the pre-Arrhenius factor and *R* is the universal gas constant. We thus obtain an activation energy *E*_a_ = 110 ± 26 kJ mol^–1^. We note that the accuracy of our results is limited here by the initial rotation value (α_0_), which depends on the rate of dissolution of glucose in water and the time elapsed to obtain a thermally equilibrated mixture.

**Figure 5:**
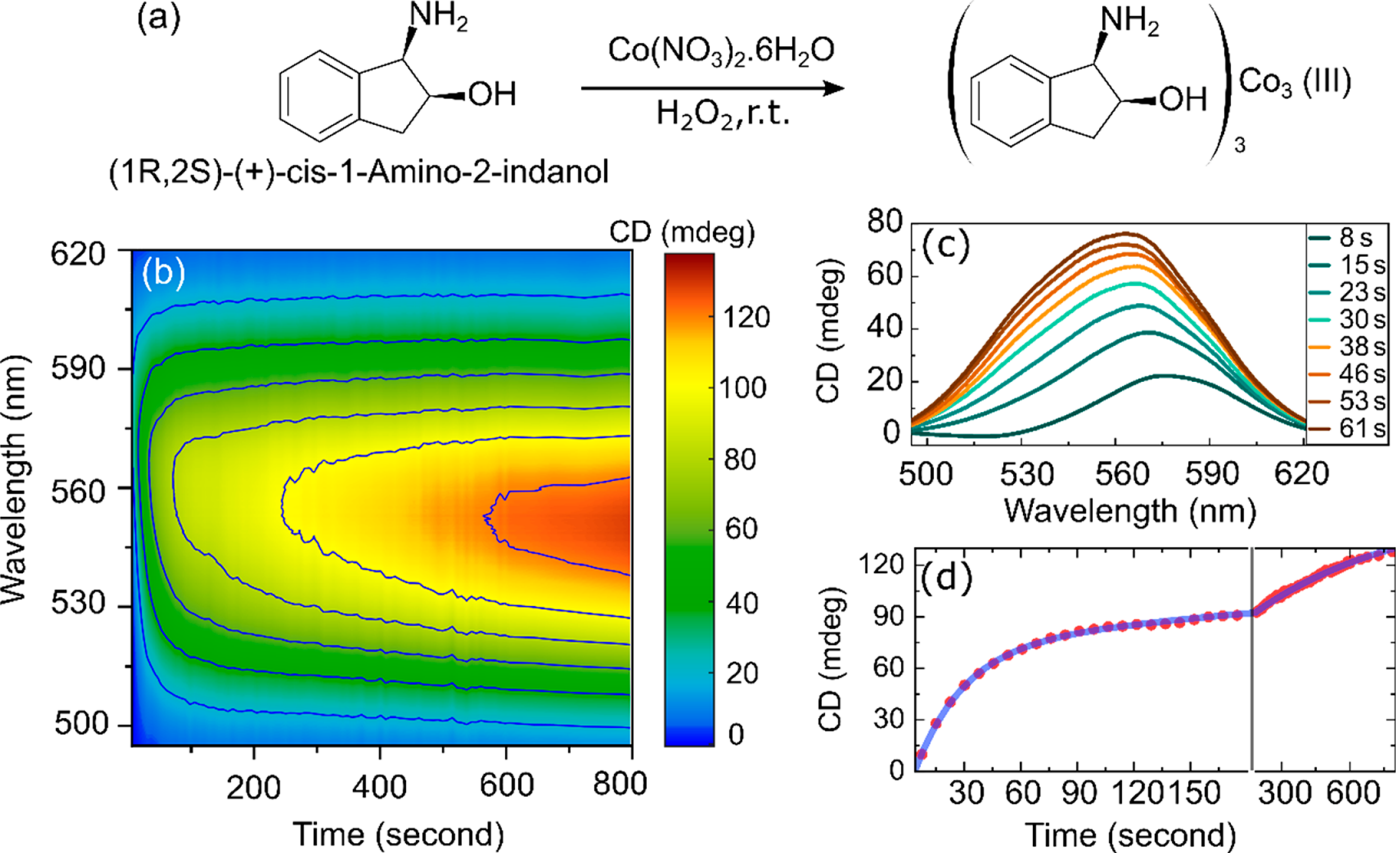
Real-time monitoring of an enantioselective chemical reaction measured in a cuvette with a 10 mm path length. (a) Formation of the chiral cobalt complex. (b) Contour plot of the evolution of CD spectra as a function of time. Each spectrum was acquired with a single scan of the interferometer, resulting in an acquisition time of ∼7 s. (c) Initial growth of the CD spectra due to chirality induction. (d) Biexponential fit of the experimental CD values at 550 nm during the chemical reaction as plotted against a linear− logarithmic time axis.

We further demonstrate the utility of our setup for optical chirality sensing applications using a cobalt complex that undergoes rapid analyte binding with chiral amino phenols.^26^ Since the reaction is accompanied by changes in absorbance, it is necessary to measure changes in chiral and achiral signals at the same time. The achiral transmission spectra are recorded simultaneously by splitting a small fraction of the light by a nonpolarizing beam splitter before the WP and measuring the intensity with a spectrometer (Figure S1). Thus, eq 7 can be rewritten as  where *Ũ*_Achi_(ω) = *f* × *T*(ω). Here, *T*(ω) is the achiral transmission spectrum recorded by the spectrometer and *f* is a fixed factor to account for the difference in achiral transmission intensity measured by the current approach to that of the previous one. The progress of the chemical reaction thus can be monitored by retrieving *Ũ*_Chi_(ω) from the balanced detection configuration while simultaneously measuring *T*(ω) with the spectrometer. Such extension of the setup therefore avoids the need to rotate the first polarizer by 45° for the measurement of achiral transmission spectra during the chemical reaction. As shown in Figure 5a, the reaction of cobalt nitrate with 1-amino-2-indanol in the presence of a catalytic amount of hydrogen peroxide is thought to produce a trimeric cobalt(III) complex. The formation of the complex is accompanied by the induction of CD signal on cobalt with a characteristic peak at 550 nm, which we track here in real-time. Figure 5b shows the contour plot of CD spectra recorded as a function of time after mixing 0.005 M Co(NO_3_)_2_ and 0.125 M (1*R*,2*S*)-(+)-*cis*-1-amino-2-indanol along with a catalytic amount of H_2_O_2_. Each spectrum was acquired with a single scan of the interferometer, resulting in an acquisition time of ∼7 s. As can be seen from Figure 5c, the formation of the chiral product becomes evident from the growth of the induced CD spectra. Thanks to the short acquisition times of our setup, a gradual shift of the spectral peak position is also discernible at early times. The time trace of the CD signal at 550 nm (Figure 5d) exhibits a biexponential rise with time constants of 26 ± 0.6 and 666 ± 28 s, respectively. Due to the fast measurement of broadband CD spectra, we are thus able to uncover a previously unknown very short kinetic time scale. These results highlight that our broadband optical setup is suitable for probing chiroptical changes during an asymmetric chemical reaction in real-time.

**Figure 6:**
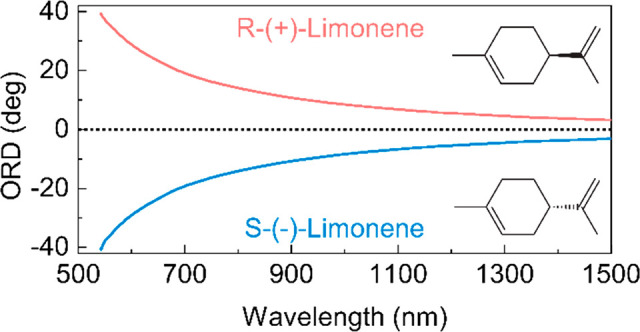
Ultrabroadband optical activity spectroscopy. ORD spectra of (*R*)- and (*S*)-limonene measured with the setup in a 10 mm path-length cuvette. The spectra were multiplied by a correction factor to account for the purity of the enantiomers.

We also extend the capability of the setup for the measurement of near-infrared vibrational optical activity. Figure 6 shows the ORD spectrum of (*R*)- and (*S*)-limonene from the visible to the near-infrared recorded using our setup, using suitable detectors. Together they cover nearly three octaves of bandwidth. Due to the extremely high interferometric stability of our CPI, the system potentially can collect spectra across the entire transparency range (190 nm to 3 μm) of the α-BBO wedges, provided that suitable broadband detectors are employed. Using other birefringent materials (such as LiNbO_3_ and Hg_2_Cl_2_), the setup can be further extended for vibrational optical activity measurements in the shortwave-IR and mid-IR range.^27,28^

## Discussion

In this paper, we have demonstrated a novel concept for the measurement of polarization rotation across a broad wavelength range by combining balanced detection with interferometric FT. Due to the extreme phase stability of the common-path interferometer, our setup enables broadband measurement of optical activity spanning the visible and near-infrared. With respect to our previous work, which relied on a strict elimination of the strong achiral signal by a nearly cross-polarized geometry,^18^ the current setup takes advantage of the strong achiral signal as the local oscillator for the heterodyne amplification. This new approach is thus akin to self-heterodyne amplification in stationary absorption or transient absorption measurements, but with access to both real and imaginary parts of the chiral signal field.

Our optical activity setup allows quick and sensitive measurement of broadband CD and ORD spectra using an incoherent thermal light source. The CD detection sensitivity is better than 2 millidegrees, corresponding to an absorptivity smaller than 6 × 10^–5^ optical density, comparable to commercial CD spectrometers but with remarkably short measurement times of just a few seconds for the full spectrum. The ORD sensitivity reaches down to 0.2 millidegree, which corresponds to a signal of 3.5 × 10^–6^ radians, comparable to the one obtained by commercial narrowband polarimeters. In contrast to the standard devices, our interferometric broadband setup relies on heterodyne amplification and phase-sensitive characterization of the chiral signal field and does not require any monochromator, photoelastic modulator, or lock-in amplifier. The common-path configuration of the setup also provides excellent interferometric stability, making it robust against surrounding disturbances and thermal changes.

The measurement of a broad spectrum only takes a few seconds and will allow for in situ identification of chiral species during a molecular aggregation^29^ and asymmetric chemical syntheses.^30^ Further reduction of the acquisition time is possible by using a brighter light source and optimizing the data transfer/processing protocols.^31^ The chiroptical sensing of a reaction, in general, is rendered difficult by interferences from various chemicals present in the reaction mixture. With the simultaneous measurement of broadband CD and ORD spectra, a phase-map of chiral signals can be generated as a function of time, from which chiral species can be identified more easily during a chemical reaction. The broadband measurements will enable users to apply advanced chemometric algorithms to isolate the desired CD spectra in the presence of other confounding substances, thus improving the sensing capability.

The sensing of glucose concentration in diabetes patients has long been a challenging goal of optical polarimetry.^32^ Glucose levels in the blood range between 70 and 130 mg/dL in normal conditions, whereas higher values indicate diabetes.^33,34^ A quantitative estimation of glucose concentration thus requires sub-millidegree levels of sensitivity. Although our setup has the required sensitivity and can detect glucose concentration as low as 20 mg/dL, it will still be challenging to accurately determine glucose concentrations in blood in the visible spectral range, where the ORD spectrum is unstructured and quite similar to the spectra of other confounding substances. The specificity of the measurements, however, could be significantly improved in the near-infrared range where the spectra are more structured.^35^

The interferometric approach demonstrated here, with its sensitivity to the electric field shifts, has a great potential for time-resolved optical activity measurements of stereochemical and structural changes.^36^ Indeed, as previously demonstrated,^37^ the CPI accepts also ultrashort broadband laser pulses, and time-domain detection will allow for the use of lasers with MHz repetition rate and high-frequency modulation of an actinic pump beam. The pump-induced changes in the chiral interferogram can be read by a high-frequency lock-in amplifier, from which broadband transient CD and ORD can be recorded with high sensitivity. Beyond spectroscopy, our approach can also be extended to CD microscopy, which so far has been limited to narrowband configurations,^38,39^ toward broadband optical activity imaging, an area that has remained largely unexplored to date.

## Conclusion

In conclusion, we have introduced a novel configuration for broadband measurements of optical activity and achieved state-of-the-art sensitivity employing an incoherent thermal light source. The key to our approach is the heterodyne amplification of weak chiral signal by the strong achiral signal and highly sensitive measurement of polarization rotation by noise-canceling balanced detection across a broad wavelength range in the time domain. We have demonstrated the potential of the setup for real-time monitoring of fast chemical reactions. It also paves the way for broadband time-resolved optical activity spectroscopy and broadband optical activity imaging with the possibility of utilizing high-repetition-rate laser sources. Since our approach of measuring broadband polarization rotation is very general, we envisage that the advantages of simplicity, affordability, and robustness of the setup will enable a wide range of experiments outside the research laboratories.

## Methods

### The Common-Path Interferometer

Our CPI is a simplified version of the translating-wedge-based identical pulses encoding system (TWINS), a birefringence-based delay line recently introduced by some of the authors.^40^ The CPI (Figure 1c) consists of two birefringent blocks made of α-barium borate. The first birefringent block (A) has two identical wedges (with apex angle 9°) with horizontal optical axes. One of the wedges is mounted on a precision translation stage, which controls its insertion along the apex angle direction and provides interferometric delay control. The second birefringent block (B) is a plane-parallel plate with a vertical optical axis and provides a fixed delay between the vertical and horizontal components. The calibration of the CPI wedge translation has been described previously in detail.^41^

## Supporting Information Available

Jones matrix analysis of the experimental setup; extension of the setup for simultaneous measurements of chiral and achiral signals (PDF)
