# Supplementary material for: Broadband Optical Activity Spectroscopy with Interferometric Fourier-Transform Balanced Detection
Source: ACS Photonics. 2021 Jul 13;8(8):2234–42. doi: 10.1021/acsphotonics.0c01866 (PMC8377715; doi:10.1021/acsphotonics.0c01866)
Supplement: Supplementary file 1 — ph0c01866_si_001.pdf [file ph0c01866_si_001.pdf]

Supporting Information

Broadband Optical Activity Spectroscopy with Interferometric Fourier-Transform Balanced Detection

Soumen Ghosh[1*], Georg Herink[2], Antonio Perri,[1,4] Fabrizio Preda,[1,4] Cristian Manzoni,[3] Dario Polli,[1,3,4] Giulio Cerullo[1,3,4*]

[1]Dipartimento di Fisica, Politecnico di Milano, Piazza Leonardo da Vinci 32, I-20133 Milano, Italy

[2]Experimental Physics VIII, University of Bayreuth, D-95447 Bayreuth, Germany

[3]Istituto di Fotonica e Nanotecnologie (IFN) - CNR, Piazza Leonardo da Vinci 32, I-20133 Milano, Italy

[4]NIREOS S.R.L., Via G. Durando 39, 20158 Milano, Italy

*Corresponding authors: soumen.ghosh@polimi.it (S.G.), giulio.cerullo@polimi.it (G.C.)

Jones matrix analysis of setup

A Jones matrix analysis of the setup (Figure 1c, main text) allows us to obtain analytical expressions for calculating CD and ORD spectra from the measured interferograms. In an (x, y) laboratory frame, let x be the horizontal axis and y be the vertical axis; a linearly polarized monochromatic light with frequency ω after the vertical polarizer can be written as:

$$E_1(\omega) = E_0(\omega)\begin{bmatrix}0\\1\end{bmatrix}$$

For an isotropic chiral sample with absorbance $\alpha = (\alpha_{\mathrm{LCP}} + \alpha_{\mathrm{RCP}})/2$, mean refractive index $n = (n_{\mathrm{LCP}} + n_{\mathrm{RCP}})/2$, and thickness L, the Jones matrix in the limit of weak CD and ORD is,

$$M_{\text{chi}} \approx 10^{-\alpha/2} \mathrm{e}^{-\mathrm{i}\rho} \begin{bmatrix} 1 & \text{ORD} - \mathrm{i}\ \text{CD} \\ -\text{ORD} + \mathrm{i}\ \text{CD} & 1 \end{bmatrix}$$

where $\text{ORD} = (n_{\text{LCP}} - n_{\text{RCP}})L\omega/(2c)$ and $\text{CD} = (\alpha_{\text{LCP}} - \alpha_{\text{RCP}}) \ln(10)/4$ are optical rotation and ellipticity in radians, $\rho = nL\omega/c$ and c is the speed of light. The field transmitted by the chiral sample is,

$$E_2(\omega) = M_{\text{chi}}\, E_1(\omega) = 10^{-\alpha/2} \mathrm{e}^{-\mathrm{i}\rho} E_0(\omega) \begin{bmatrix} \text{ORD} - \mathrm{i}\ \text{CD} \\ 1 \end{bmatrix}$$

The CPI introduces relative variable retardation between the y-polarized component and the x-polarized component,

$$E_3(\omega, \tau) = 10^{-\alpha/2}\ \mathrm{e}^{-\mathrm{i}\rho}\ E_0(\omega) \begin{bmatrix} \text{ORD} - \mathrm{i}\ \text{CD} \\ \mathrm{e}^{\mathrm{i}\phi} \end{bmatrix}$$

where $\phi = \omega \Delta n(\omega) l/c$ is the phase difference introduced between horizontal and vertically polarized components; in particular $\Delta n = |n_o - n_e|$ is the birefringence of α-BBO, and l is the effective thickness of birefringent material traversed by the beam. For simplicity, we can consider $\phi = \omega\tau$ where τ is the relative delay between the two orthogonal components imparted by the CPI.

The Wollaston prism oriented at 45° to the vertical can be considered as two polarizers oriented at ±45°. Hence the output at two channels of the WP becomes,

$$E_4^{+}(\omega, \tau) = \frac{1}{2} \begin{bmatrix} 1 & 1 \\ 1 & 1 \end{bmatrix} E_3(\omega, \tau) = \frac{1}{2}.10^{-\alpha/2}\ \mathrm{e}^{-\mathrm{i}\rho}\ E_0(\omega) \begin{bmatrix} \text{ORD} - i\ \text{CD} + \mathrm{e}^{\mathrm{i}\phi} \\ \text{ORD} - i\ \text{CD} + \mathrm{e}^{\mathrm{i}\phi} \end{bmatrix}$$

$$E_4^{-}(\omega, \tau) = \frac{1}{2} \begin{bmatrix} 1 & -1 \\ -1 & 1 \end{bmatrix} E_3(\omega, \tau) = \frac{1}{2}.10^{-\alpha/2}\ \mathrm{e}^{-\mathrm{i}\rho}\ E_0(\omega) \begin{bmatrix} \text{ORD} - i\ \text{CD} - \mathrm{e}^{\mathrm{i}\phi} \\ -\text{ORD} + i\ \text{CD} + \mathrm{e}^{\mathrm{i}\phi} \end{bmatrix}$$

By calculating the signal as a function of the CPI delay and integrating over all the frequency components, the output intensity at the two channels of the BPD is,

$$I_4^+(\tau) = \int \mathrm{d}\omega \ |E_4^+(\omega,\tau)|^2 = \int \mathrm{d}\omega \ |E_0(\omega)|^2 \ \frac{1}{2}.10^{-\alpha(\omega)}[1 + 2\mathrm{ORD}\cos(\omega\tau) - 2\mathrm{CD}\sin(\omega\tau)]$$

$$I_4^-(\tau) = \int \mathrm{d}\omega \ |E_4^-(\omega,\tau)|^2 = \int \mathrm{d}\omega \ |E_0(\omega)|^2 \ \frac{1}{2}.10^{-\alpha(\omega)}[1 - 2\mathrm{ORD}\cos(\omega\tau) + 2\mathrm{CD}\sin(\omega\tau)]$$

Thus, the differential output intensity of the chiral interferogram is,

$$I_{\mathrm{chi}}(\tau) = I_4^+(\tau) - I_4^-(\tau) = 2\int \mathrm{d}\omega \ |E_0(\omega)|^2 \ 10^{-\alpha(\omega)}[\mathrm{ORD}\cos(\omega\tau) - \mathrm{CD}\sin(\omega\tau)]$$

The above expression contains the heterodyne amplified signal as a product between the transmitted light field and the CFID, where imaginary and real parts of CFID correspond to the CD and ORD values, respectively. To extract the CFID only part, a calibration achiral interferogram using the autocorrelation of the transmitted achiral field needs to be recorded. This is done by rotating the first polarizer by 45°; the resulting interferogram is:

$$I_{\mathrm{Achi}}(\tau) = \int \mathrm{d}\omega \ |E_0(\omega)|^2 \ 10^{-\alpha(\omega)}\cos(\omega\tau)$$

Notice the shift in phase between calibration and chiral interferogram, which depends on the strength of the CD signal. Both CD and ORD spectra are recovered by taking the ratio of the Fourier-transforms of the chiral and calibration interferograms,

$$\mathrm{CD}(\omega) = -\frac{1}{2}\ \mathrm{Im}\left[\frac{FT[I_{\mathrm{chi}}(\tau)]}{FT[I_{\mathrm{cal}}(\tau)]}\right]$$

$$\mathrm{ORD}(\omega) = \frac{1}{2}\ \mathrm{Re}\left[\frac{FT[I_{\mathrm{chi}}(\tau)]}{FT[I_{\mathrm{cal}}(\tau)]}\right]$$

Extension of the setup for the simultaneous measurement of chiral and achiral signals

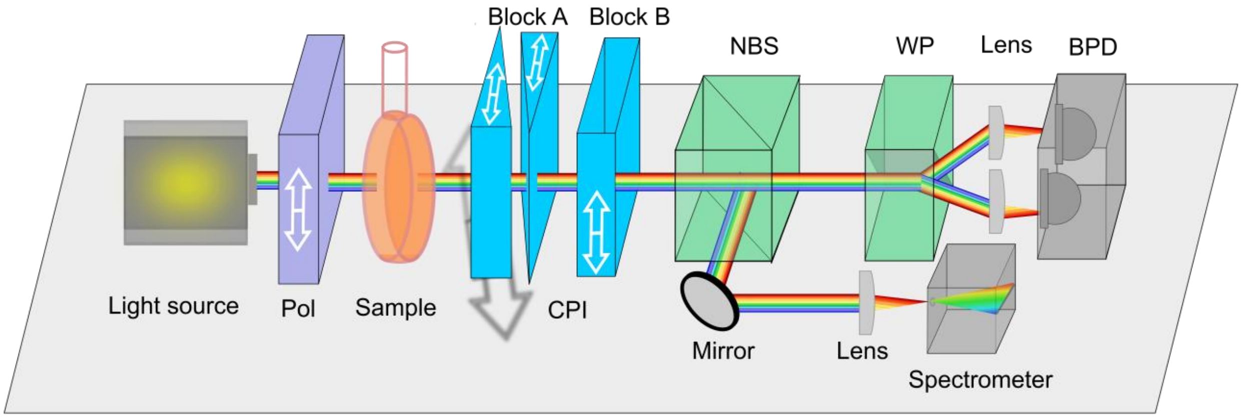

Figure S1. Extended experimental setup for the simultaneous measurements of chiral and achiral signals. Pol: Polarizer; CPI: Common-path interferometer; NBS: Non-polarizing beam splitter; WP: Wollaston prism; BPD: Balanced photodetector. The white double arrows indicate the optical axes of the polarization optics. The grey double-arrow indicates the direction of the wedge translation.
